# The power and interest indicators of the stakeholders of a Water User Association around Bengawan Solo River, Indonesia

**DOI:** 10.1016/j.dib.2018.07.030

**Published:** 2018-07-18

**Authors:** Rustinsyah Rustinsyah

**Affiliations:** Airlangga University, Indonesia

**Keywords:** Stakeholder, Power, Interest, Agricultural irrigation management

## Abstract

This data article presents the information on stakeholders’ power and interest indicators in agricultural irrigation management. The data were collected from a WUA (Water User Association) called *Sekar padi* which operates around Bengawan Solo river, East Java, Indonesia. This data article contains two major data. The first data consists of the power indicator from both primary and secondary stakeholders of WUAs. The second data is the stakeholders ‘interest indicator of WUAs in agricultural irrigation management. The data were collected from observations, Focus Group Discussion (FGD) and interview. These data will be beneficial for policy makers to determine the suitable programs for agricultural irrigation management and for researchers who want to conduct similar studies in developing countries.

**Specifications Table**

**Table:** 

Subject area	*Social Sciences*
More specific subject area	*Rural studies*
Type of data	*Tables*
How data was acquired	*observations, focus group discussions, and interview using interview guidelines.*
Data format	*Raw and analysed*
Experimental factors	*A participatory observation was conducted prior to the FGD and the interview.*
Experimental features	*1. Participatory observations*
	*2. Focus Group Discussion*
	*3. Interviewing the stakeholders individually to understand the levels of power and interest.*
Data source location	*Bandungrejo Village, Tuban regency, East Java, Indonesia.*
Data accessibility	*The data is included in this article*

**Values of the data**

• The knowledge in the power indicators of each type of stakeholder and the level of category of the stakeholders will be beneficial in determining the right stakeholder for implementing a particular agricultural irrigation program.
• The knowledge in the interest indicators of WUA׳s stakeholders will enable the formulation of the right programs for agricultural irrigation management.
• The data on power and interest indicators presented in this study can be used as comparison for the data obtained from similar studies conducted in other developing countries.
• Researcher from the social sciences will be able to draw from the power and interest indicators to conduct other studies related to agricultural irrigation management.

## 1 Data

There are two main data presented in this article. The first data is a stakeholder power indicator in agricultural irrigation management. Stakeholders are influencing or being influenced individuals or groups to achieve certain goals. Freeman [3] believes that stakeholders have position, power, and interest related to certain intention. Moreover, Grimble and Wellard [4] also claim that there are authoritative linkages between the power and the type of stakeholders. According to Morgenthau [5], power is also a major goal of policy or even a determining motive of any political action. The power indicator is related to authority and networking [2]. In this paper, the power indicators of WUAs stakeholder can be categorised into four indicators, they are (1) authority, (2) capability and capacity, (3) credibility, (4) networking. These indicators are determined in relation to the type of stakeholder, i.e. primary or secondary, in carrying out the tasks and obligations in managing agricultural irrigation in the village. In addition, there are also levels of categories of stakeholders. In this case, the levels are categorized into very high, high, fairly high, and low. The tabulation of the power indicator of the stakeholders of Sekarpadi WUA is presented in Table 1.

**Table 1:** Stakeholders’ power indicators in agricultural irrigation management.

**No**	**Stakeholders**	**Type of Stakeholders**	**Power Indicators**					**Level of Category**
			**Authority**	**Capability**	**Credibility**	**Capacity**	**Mass Mobilization**	
1	Head of Village	Primary	√	√	√	√	√	Very High
2	WUA Leader		√	√	√	√	√	Very High
3	Secretary	Primary	√	√	√	√	√	Very High
4	Treasurer	Primary	√	√	√	√	√	Very High
5	Technical Officer	Primary	√	√	√	√	–	High
6	Area Coordinator 1	Primary	√	√	√	√	–	High
7	Area Coordinator 2	Primary	√	√	√	√	–	High
8	Area Coordinator 3	Primary	√	√	√	√	–	High
9	Area Coordinator 4	Primary	√	√	√	√	–	High
10	Work Group 1	Primary	√	√	√	√	–	High
11	Work Group 2	Primary	√	√	√	√	–	High
12	Work Group 3	Primary	√	√	√	√	–	High
13	Work Group 4	Primary	√	√	√	√	–	High
14	Work Group 5	Primary	√	√	√	√	–	High
15	Work Group 6	Primary	√	√	√	√	–	High
16	Work Group 7	Primary	√	√	√	√	–	High
17	Work Group 8	Primary	√	√	√	√	–	High
18	Work Group 9	Primary	√	√	√	√	–	High
19	Work Group 10	Primary	√	√	√	√	–	High
20	Work Group 11	Primary	√	√	√	√	–	High
21	Work Group 12	Primary	√	√	√	√	–	High
22	Work Group 13	Primary	√	√	√	√	–	High
23	Work Group 14	Primary	√	√	√	√	–	High
24	Work Group 15	Primary	√	√	√	√	–	High
25	Work Group 16	Primary	√	√	√	√	–	High
26	Work Group 17	Primary	√	√	√	√	–	High
27	Work Group 18	Primary	√	√	√	√	–	High
28	Work Group 19	Primary	√	√	√	√	–	High
29	Work Group 20	Primary	√	√	√	√	–	High
30	Work Group 21	Primary	√	√	√	√	–	High
31	Work Group 23	Primary	√	√	√	√	–	High
32	Work Group 24	Primary	√	√	√	√	–	High
33	Operator and Driver 1	Primary	√	√	√	√	–	High
34	Operator and Driver 2	Primary	√	√	√	√	–	High
35	Operator and Driver 3	Primary	√	√	√	√	–	High
36	Supervisory Body 1	Primary	√	√	√	√	√	Very High
37	Supervisory Body 2	Primary	√	√	√	√	√	Very High
38	Supervisory Body 3	Primary	√	√	√	√	√	Very High
39	WUA Member	Primary	√	√	–	–	√	High
40	District-Level Advisory Body	Secondary	√	–	–	–	–	Fairly High
41	Public Figure	Secondary	–	–	√	–	√	Fairly High
42	Village Apparatus	Secondary	√	√	–	–	–	Fairly High
43	Farmer Group	Secondary	√	√	–	–	–	Fairly High
44	Agricultural Product Buyer	Secondary	√	√	–	–	–	Fairly High
45	Office of Public Works (Water Resources Division)	Secondary	√	–	√	–	–	Fairly High
46	Office of Agriculture	Secondary	√	–	√	–	–	Fairly High
47	Office of Bengawan Solo River Water Management	Secondary	–	–	–	–	–	Low
48	Worker	Secondary	–	–	–	–	–	Low
49	Food Stall Owner	Secondary	–	–	–	–	–	Low
50	Fertilizer and Farm Medicine Shop Owner	Secondary	–	–	–	–	–	Low

Description:

• *Authority* is the right to take action or right to make rules to govern others.
• *Capability and capacity* are measures of the ability of an entity (i.e. department, organization, people) to achieve its objectives, especially in relation to the overall mission.
• *Credibility* is a power to generate trust.
• *Networking* is a useful and mutually beneficial relationship.

1) Primary Stakeholders

*Very High-Power Stakeholder*:

a) Informants Number 1- as a chairman, democratically elected by the villagers.
b) Informants Number 2–9 as the core management with qualified authority, capability, credibility and capacity, democratically elected by HIPPA members, for managing the village׳s agricultural irrigation and rice farming activities matters.
c) Informants Number 36–38 as supervisory members who are democratically elected to provide consultations.

*High Power Stakeholder*

a) Informants Number 10–35 have the capability, credibility, capacity to assist the core management in managing agricultural irrigation. They convey aspirations and problems (i.e. floods, water supplies delay and others) from HIPPA members to the core management to get immediate response or problem solving.
b) Informant Number 39 is a member with capability and capacity to assess HIPPA׳s member performance at accountability report meeting. For the example: Accountability report cannot be accepted when unresolved issue arises (i.e. financial problems).

2) *Secondary Stakeholders*

*Fairly High Power*

Informants Number 40–47 do not intervene in water distribution management, but they have capacity in solving water management problems. For the examples: (1) public works service department and water resources sub-field department support in irrigation infrastructure development, (2) department of Agriculture through association of farmers group in villages assist farming activities (i.e. Distributing subsidized fertilizer, eradicating pest and so on).

*Low Power*

Informants Numbers 48–50 do not have power over HIPPA in agricultural irrigation management. However, they can give suggestions related to water distributions finding issues and rice farming.

The second data present the interest indicators of WUA׳s stakeholder. According to Bryson [1], interest is the will and desire of a person or a group for an activity. The interest indicators of WUA׳s stakeholders can be categorized into three: (1) hope, (2) reputation, and (3) potential benefit. Similar to the stakeholders’ power indicator, there are also levels of categories, which in this case is called “degree of interest”. The levels are very high, high, fairly high, and low. The stakeholders’ interest indicator tabulation in agricultural irrigation management is shown in Table 2.

**Table 2:** Stakeholders’ interest indicators in agricultural irrigation management.

**No**	**Codes**	**Types of Stakeholder**	**Interest Indicators**			**Degree of Interest**
			**Hope**	**Aspiration**	**Potential Benefit**	
1.	Head of Village	Primary	Very High	Very High	Very High	Very High
2.	WUAs Leader	Primary	Very High	Very High	Very High	Very High
3.	Secretary	Primary	High	High	High	High
4.	Treasurer	Primary	High	High	High	High
5.	Technical	Primary	High	High	High	High
6.	Area Coordinator 1	Primary	High	High	High	High
7.	Area Coordinator 2	Primary	High	High	High	High
8.	Area Coordinator 3	Primary	High	High	High	High
9.	Area Coordinator 4	Primary	High	High	High	High
10.	Head of Work Group 1	Primary	High	High	High	High
11.	Head of Work Group 2	Primary	High	High	High	High
12.	Head of Work Group 3	Primary	High	High	High	High
13.	Head of Work Group 4	Primary	High	High	High	High
14.	Head of Work Group 5	Primary	High	High	High	High
15.	Head of Work Group 6	Primary	High	High	High	High
16.	Head of Work Group 7	Primary	High	High	High	High
17	Head of Work Group 8	Primary	High	High	High	High
18	Head of Work Group 9	Primary	High	High	High	High
19	Head of Work Group 10	Primary	High	High	High	High
20.	Head of Work Group 11	Primary	High	High	High	High
21	Head of Work Group 12	Primary	High	High	High	High
22.	Head of Work Group 13	Primary	High	High	High	High
23.	Head of Work Group 14	Primary	High	High	High	High
24	Head of Work Group 15	Primary	High	High	High	High
25	Head of Work Group 16	Primary	High	High	High	High
26.	Head of Work Group 17	Primary	High	High	High	High
27.	Head of Work Group 18	Primary	High	High	High	High
28	Head of Work Group 19	Primary	High	High	High	High
29.	Head of Work Group 20	Primary	High	High	High	High
30.	Head of Work Group 21	Primary	High	High	High	High
31	Head of Work Group 22	Primary	High	High	High	High
32.	Head of Work Group 23	Primary	High	High	High	High
33.	Operator and Driver	Primary	High	High	High	High
34.	Operator and Driver	Primary	High	High	High	High
35	Operator and Driver	Primary	High	High	High	High
36	Supervisory Body	Primary	High	High	High	High
37.	Supervisory Body	Primary	High	High	High	High
38.	Supervisory Body	Primary	High	High	High	High
39	WUAs Member	Primary	High	High	High	High
40	District-Level Advisory Body	Secondary	High	High	Fairly High	Fairly High
41	Public Figure	Secondary	High	High	Fairly High	Fairly High
42	Village Apparatus	Secondary	High	High	Fairly High	Fairly High
43	Farmer Group	Secondary	High	High	High	High
44.	Agricultural Product Buyer	Secondary	High	High	High	High
45.	Office of Public Works	Secondary	High	High	High	High
46	Office of Agriculture	Secondary	High	High	High	High
47.	Office of Bengawan Solo River Water Management	Primary	Fairly High	Fairly High	Fairly High	Fairly High
48	Worker	Secondary	High	High	High	High
49.	Food Stall Owner	Secondary	High	Fairly High	Low	Fairly High
50.	Agricultural Drug Shop Owner	Secondary	High	High	High	High

## 2 Experimental design, materials and methods

There are six WUAs in the district that use the Bengawan Solo River water for agricultural irrigation. These six WUAs are: a) WUA in Bandungrejo Village; b) WUA in Plandirejo Village; c) WUA in Klotok Village; d) WUA in Kedungrejo Village; e) WUA in Magersari Village; and f) WUA in Plumpang Village. The data presented in this article is from the most experienced and successful WUA, i.e. Sekarpadi WUA in Bandungrejo Village. There were three steps taken to collect the data.

First, participatory observations were conducted on the work system of the WUA in agricultural irrigation management, which includes the water distribution system, irrigation network, and stakeholder performance to overcome the problems faced by HIPPA in agricultural irrigation management to success in farm cultivating. Second, conducting Focus Group Discussion (FGD) among HIPPA׳s members. Third, conducting individual interviews using the interview guidelines (Appendix). Interview guidelines for FGDs are directed to know, and understand the issues faced by HIPA in managing agricultural irrigations. It was also aimed at knowing who has the power (authority, capability, credibility, capacity, and networking) to solve the problems, and what the strategies are to solve the problems.

The data collected were then classified based on the power and interest levels of each stakeholder. To ease the readability of the collected data, the tabulation of the data was presented in the form of tables (see Table 1 and Table 2). These data will be beneficial for policy makers and researchers to know the conditions of the power and interest of the people in this rural area. The data can also be used as the basis for conducting similar research in other villages in developing countries.

## Appendix: Interview Guidelines

• Questions related to the Power Indicators

1. What have been done by HIPPA to make the farming in this village become more successful?
2. What were the problems faced by HIPPA in managing the agricultural irrigation in this village?
3. What were the strategies used by HIPPA to solve the problems?
4. Who has the highest power in overcoming each of the problems?

• Questions related to the Interest Indicators

1. What motivated the stakeholders to work with HIPPA?
2. What benefits have the stakeholders gained after working with HIPPA?
