# Supplementary material for: The power and interest indicators of the stakeholders of a Water User Association around Bengawan Solo River, Indonesia
Source: Data Brief. 2018 Jul 18;19:2398–403. doi: 10.1016/j.dib.2018.07.030 (PMC6141517; doi:10.1016/j.dib.2018.07.030)
Supplement: Supplementary file 1 — Supplementary material [file mmc1.docx]

**AUTHOR DECLARATION**

I wish to confirm that there are no known conflicts of interest associated with this publication and there has been no significant financial support for this work that could have influenced its outcome.

I confirm that there are no other persons who satisfied the criteria for authorship but are not listed.

I confirm that I have given due consideration to the protection of intellectual property associated with this work and that there are no impediments to publication, including the timing of publication, with respect to intellectual property. In so doing I confirm that I have followed the regulations of my institutions concerning intellectual property.

I understand that the Corresponding Author is the sole contact for the Editorial process (including Editorial Manager and direct communications with the office). She is responsible for communicating with submissions of revisions and final approval of proofs. I confirm that I have provided a current, correct email address which is accessible by the Corresponding Author and which has been configured to accept email from rustinsyah58@yahoo.com

Indonesia, 15 May 2019


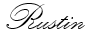


Dr. Rustinsyah
